# Leprosy and tuberculosis control scenario of the national program for the improvement of access and quality of primary care in Brazil

**DOI:** 10.1186/s12913-023-09842-5

**Published:** 2023-08-02

**Authors:** Glenda R. O. N. Ferreira, Amanda L. C. Miranda, Viviane A. Farias, Melissa B. Martins, Débora Talitha Neri, William D. Borges, Carlos Leonardo F. Cunha, Geyse Aline R. Dias, Dirceu C. Santos, Fabianne J. D. Sousa

**Affiliations:** 1 Programa de Pós Graduação em Enfermagem, Federal University of Para, Rua Augusto Correa, 01 – Setor Saúde. Guamá, Belém, Pará 66075-110 Brazil; 2 Universidade da Amazônia, Belém, Pará Brazil

**Keywords:** Primary Health Care, Tuberculosis, Leprosy, Communicable disease control, Health Evaluation

## Abstract

**Background:**

In Brazil, despite advances in public health policies aimed at eliminating and controlling infectious and parasitic diseases, the incidence of neglected diseases is still high. The epidemiological scenario in Brazil of diseases such as tuberculosis and leprosy evidences a public policy agenda that has not been resolute in terms of control, nor in terms of elimination.

**Objective:**

To analyze the actions of diagnosis and treatment of leprosy and tuberculosis in the context of primary health care.

**Methods:**

In this ecological study, data from the third cycle of the Program for the Improvement of Access and Quality of Primary Care were extracted from electronic address of the Primary Health Care Secretariat of Brazil in the area of Actions, Programs and Strategies. A total of 37,350 primary health care teams were that answered the questionnaire were eligible, with variables extracted from leprosy and tuberculosis control actions. The municipalities were grouped according to the characteristic of the Brazilian municipality. The partition chi-square and the Residuals Test were used to assess whether there was a difference in the proportion of tuberculosis and leprosy actions between types of municipalities. Statistics were carried out using Minitab 20 and Bioestat 5.3.

**Results:**

Regarding the leprosy treatment location, there is a higher proportion of people referred to be treated at the reference in adjacent rural (*p* = 0.0097) and urban (*p* < 0.0001) municipalities; monitoring of people with leprosy referred to the service network (*p*. = 0.0057) in remote rural areas. Lower proportion of teams requesting bacilloscopy in remote rural areas (*p* = 0.0019). Rural areas have a higher proportion of teams that diagnose new cases (*p* = 0.0004). Regarding the actions of diagnosis and treatment of tuberculosis. There is a higher proportion of teams that carry out consultations at the unit itself in rural areas when compared to adjacent intermediaries (*p* = 0.0099) and urban (*p* < 0.0001); who requested sputum smear microscopy in adjacent intermediaries (*p* = 0.0021); X-ray in adjacent intermediaries (*p* < 0.0001) and urban (*p* < 0.0001); collection of the first sputum sample in urban (*p* < 0.0001) and adjacent rural areas (*p* < 0.0001); directly observed treatment (*p* < 0.0001) in adjacent rural municipalities.

**Conclusion:**

There are inequalities in the diagnosis and treatment of leprosy and tuberculosis among the types of municipalities.

## Introduction

In Brazil despite advances in public health policies aimed at eliminating and controlling infectious and parasitic diseases, the incidence of neglected diseases is still high [1]. This country was included in the list of the 30 countries with the highest number of Tuberculosis cases. But among these countries, Brazil has the highest levels of treatment coverage in 2021, along with Bangladesh, China, Uganda and Zambia [2]. Leprosy is a neglected disease that led the country to be included in the list of 23 priority ones, however, the situation is even more serious, Brazil, India and Indonesia accounted for 74.5% of the new leprosy cases detected worldwide in 2021 [3]. In the world, in 2021, the incidence of Tuberculosis was 6.4 million cases, with 1.4 million deaths [2], while for leprosy there were 140,594 cases [3]. In Brazil, there were 69, 271 new cases of Tuberculosis and 18,318 cases of leprosy [3, 4].

These diseases associated with vulnerability, poverty and low income [1–4], whose worldwide distribution allows identifying the cartography of this inequality [1]. This epidemiological scenario prioritized the inclusion of these diseases in the global agenda of the Sustainable Development Goals. The goal of ending these diseases by 2030 can be achieved with universal access to health and intersectoral actions on social determinants [1–6]. These are preventable and treatable diseases within the scope of Primary Health Care. In Brazil, primary health care teams carry out prevention, diagnosis and treatment actions for tuberculosis and leprosy in the health unit itself, with imaging and laboratory tests performed in other services of the health network [5, 6].

In Brazil, in relation to leprosy, in the Basic Health Unit there is still a lack of basic resources for diagnosis and treatment, in addition to failures in the surveillance of contacts [7–9]. Tuberculosis prevention, diagnosis and treatment actions also have problems related to the attributes of the primary health care, with barriers to access to diagnosis, structure, recording instruments and the need to advance in the articulation between the health care points. The failures that exist in processes related to directly observed treatment have an impact on the results of cure, abandonment and death [10]. Furthermore, the biomedical care model still persists, it is present even among the Family Health Strategy teams, lack of link between the health professional and the person [11].

These deficiencies in the structure of the Basic Health Unit and in the processes of the Primary Health Care teams can be analyzed based on data from the National Program for the Improvement of Access and Quality of Primary Care (PMAQ-AB in portuguese). In addition, it also makes it possible to geographically analyze the distribution of Basic Health Units, demonstrating the heterogeneity that exists between municipalities and between geographic regions [9, 11–14]. For diseases such as Tuberculosis and leprosy, the high values of the outcome indicators indicate failures in health care and they also reflect social inequalities [1–4, 15].

The epidemiological scenario of these diseases in Brazil evidences a public policy agenda that has not been resolute in terms of control, nor in terms of elimination. However, it is capable of pointing out the marked regional inequalities that exist even among urban municipalities [4, 15–17]. In this perspective, the analysis of the Program for the Improvement of Access and Quality of Primary Care evaluation data provides subsidies for the health system management actions [14]. Which leads to the following question: the data of the actions of diagnosis and treatment of Tuberculosis and leprosy, evaluated by the Program for Improvement of Access and Quality of Primary Care, differ between the Primary Health Care teams that work in the municipalities classified as remote rural, adjacent rural, remote intermediate, adjacent intermediate and urban?

These five (05) typologies described above are the result of different criteria for classifying Brazilian municipalities, proposed by the Brazilian Institute of Geography and Statistics. In this classification, the criteria of population in areas of dense occupation, proportion of population in areas of dense occupation in relation to the total population and location were used [16]. Until the literature review stage, this is the first study to analyze data on the structure and process of Tuberculosis and leprosy control actions that were evaluated by the Program for the Improvement of Access and Quality of Primary Care considering the typology of municipalities in rural and urban spaces. Previous studies carried out with data from the Program for the Improvement of Access and Quality of Primary Care associated the population size of the municipalities [18], the regions [19] and health indicators [12] associated with care actions for people with Tuberculosis [12, 18, 19]. In this context, the evaluation of data on Tuberculosis and leprosy actions of diagnosis and treatment associated with the new typology of municipalities is relevant because it brings evidence of the current situation of municipalities and, consequently, makes it possible to compare with the impacts of the Primary Health Care financing policy that has different weights for the transfer of resources according to typology. Thus, the objective of this study was to analyze the actions of diagnosis and treatment of leprosy and tuberculosis in the context of primary health care, based on data from the National Program for the Improvement of Access and Quality of Primary Care.

## Methods

### Study design

This ecological study was conducted in Brazil, with administrative data from the third cycle of the Program for the Improvement of Access and Quality of Primary Care, coordinated by the Brazilian Ministry of Health, from 2017 to 2018. The first cycle corresponded to the adherence and contractualization phase, in which all primary health care teams could adhere. The second cycle was certification, consisting of external evaluation of the performance of health teams and primary care management; performance evaluation of contractualized indicators in the adhesion and contractualization stage; self-assessment moment. The third cycle was the re-contractualization, a singular agreement with the increment of new standards and quality indicators, stimulating the institutionalization of a cyclical and systematic process based on the results verified in the previous phase [20].

### Setting

Data from 28,897 Basic Health Units teams were used, corresponding to 63.43% of those existing in the Health Unic System (SUS, in Portugese) in January 2017 (45,557). They are distributed in 5,310 participating municipalities, with 433 located in the North region, 466 located in the Midwest region, 1,083 in the South, 1,573 in the Southeast and 1,755 in the Northeast [16, 20].

This typology was defined according to a process of classifications and matrix crossings [16]:

- Predominantly urban municipality: 1) municipalities in Population Units with more than 50,000 inhabitants in densely populated areas; 2) that have between 25,000 and 50,000 inhabitants in densely occupied areas with a degree of urbanization greater than 50%; 3) that have between 10,000 and 25,000 inhabitants in densely populated areas with a degree of urbanization greater than 75% [16].
- Intermediate municipality: 1) municipalities in Population Units that have between 25,000 and 50,000 inhabitants in densely populated areas with a degree of urbanization between 25 and 50%; 2) that have between 10,000 and 25,000 inhabitants in densely populated areas with a degree of urbanization between 50 and 75%; 3) that have between 3,000 and 10,000 inhabitants in densely populated areas with a degree of urbanization greater than 75% [16].
- Predominantly rural municipality: 1) municipalities in Population Units that have between 25,000 and 50,000 inhabitants in densely populated areas with less than 25% urbanization; 2) that have between 10,000 and 25,000 inhabitants in densely populated areas with a degree of urbanization below 50%; 3) that have between 3,000 and 10,000 inhabitants in densely populated areas with a degree of urbanization below 75% [16].
- The dimension of location distinguishes, among municipalities classified as intermediate and rural, those adjacent to higher-ranking urban centers from those that are remote [16].

### Data sources

Data collection took place in January 2020. The data were extracted from the electronic address of the Primary Health Care Secretariat in the area of Actions, Programs and Strategies, provided by the Brazilian Ministry of Health [20] and Brazilian Institute of Geography and Statistics [16].

The data source was the third cycle questionnaire, extracting the data that evaluated the actions carried out by primary health care focused on leprosy and tuberculosis. The questionnaire was answered by the primary health care teams that voluntarily joined the program [14, 20]. The present study used data from module two 1) data on care for people with leprosy and Tuberculosis and 2) the relationship between Primary Health Care and other points of the health care network.

The data of classification of municipalities were from the Brazilian Institute of Geography and Statistics: geocode, name of the municipality and classification into rural and urban spaces [16].

### Study size

The target population was the team that works in the Primary Health Care, with an intentional sampling of the municipalities that re-contracted. A total of 37,350 teams were eligible for the study who responded to module II—interview with the Primary Health Care Team professional and verification of documents at the Basic Health Unit. Excluded 1,515 whose questionnaire was not applied and four unclassified municipalities corresponding to eight teams.

### Variables

The independent variables of the study were the nomenclature of the classification of municipalities: remote rural, adjacent rural, remote intermediate, adjacent intermediate and urban.

The chosen dependent variables are part of module II, item II.1—General identification and item II.2. Identification of the Health Unit, in addition to the specific variables, described in Table 1 [20].

**Table 1:** Variables (code and description) of care for people with Tuberculosis and leprosy

II.12—PC relationship with other points of the Health Care Network
Which of these tests are requested by your team to be performed in the health services network? II.12.2.1^a^—Sputum smear microscopy for the diagnosis of tuberculosis; II.12.2.2^a^ —Bacilloscopy for leprosy; II.12.2.10^a^ —Chest X-ray—Tuberculosis^a^
II.20—Care for people with Tuberculosis II.20.1^a^ —Does the primary care team have a record of the number of people with tuberculosis? II.20.2^b^—When there is a person diagnosed with Tuberculosis, the team. II.20.4—General^c^—Is the first Sputum smear microscopy for the diagnosis of Tuberculosis collected in the first approach/consultation? II.20.5—General^c^—Does the team notify people with tuberculosis diagnosed in the unit? II.20.6^c^—Does the team monitor the treatment directly observed from the user? II.20.7.1^c^—Respiratory symptoms; II.20.7.2^c^—contact person; II.20.7.4^c^—person lost to follow-up ( Treatment abandonment)- after a period of 30 days
II.21—Care for people with leprosy II.21.1^a^ Does the primary care team have a record of the number of people with leprosy? II.21.2^b^ When there is a person diagnosed with leprosy the team. II.21.3 – General^c^ Does the team diagnose new cases of leprosy? II.21.5—General^c^ Do you notify the leprosy diagnoses made in the unit? II.21.6^c^ Does the team monitor a person referred to reference health services? II.21.7.1^c^ Symptomatic (skin lesions); II.21.7.2 ^c^ contact person; II.21.7.4^c^ Treatment abandonment

^a^answer: Yes and No

^b^answer: Conduct consultation in the unit itself or Refers the person to the reference unit

^c^answer: yes, no, not applicable

### Statistical methods

The relationship between the classification of municipalitie**s** and Program for the Improvement of Access and Quality of Primary Care databases was performed using the geocode of the municipality existing in both databases, using the PROC V function of the Microsoft Excel program. The veracity of each pair found was performed by double checking the variables: name of the municipality and the state. This program was also used to calculate absolute and relative frequencies. Answers “not applicable” were not considered in the analyses.

The chi-square test was used to assess whether there was a difference in the proportion of tuberculosis and leprosy actions between types of municipalities. The municipalities were classified according to the urban rural typology, following order: rural remote, rural adjacent, intermediate remote, intermediate adjacent and urban, recording in each one the action regarding execution.

The rural municipalities were compared with each other and then the other typologies were compared. The partition chi-square test was applied. From a 2 × 5 contingency table, partition is made into others of size 2 × 2, each with one degree of freedom. The division begins at the top left corner of the general table, combining rows and columns until the whole is covered. The proportion of the first two columns are compared (remote rural and adjacent rural) to verify if there is a statistical difference, then the proportion of these is compared with each of the other columns (remote intermediate, adjacent intermediate and urban). The partition result allows identifying in which of the tested sets the statistical association occurred [21, 22].

Finally, for the variables that showed statistical significance, the Residuals Test was used. This collaborates in the interpretation of the Chi-Square by determining the probabilistic importance of each of the results, complementing the Partition Test with more specificity, as it is possible to compare the results of each value obtained with the standardized probability of the normal curve, since the denominator of the final Residuals equation is comparable to the standard error [21, 22]. In this last analysis, an adjusted residual value equal to or greater than 1.96 was considered for a *p* < 0.05. These analyzes were performed using the bioestat® program. The Minitab 20 program was used to carry out exploratory data analysis, with calculation of mean and confidence interval. Data were presented in tables.

## Results

Thirty-seven thousand three hundred forty-two teams responded to the questionnaire, 56% (20,923) of the teams were from urban municipalities, 30% (11,190) from adjacent rural areas, 10% (3,752) from adjacent intermediaries, 3.1% (1,151) from remote rural areas, 0.9% (326) of remote intermediaries.

Table 2 demonstrates that the place of treatment of the person diagnosed with leprosy in remote rural and adjacent rural municipalities presents a statistically significant difference (*p* = 0.0097). Showing a higher proportion of treatment in the reference of adjacent rural municipalities. When rural municipalities are compared with the place of treatment in urban municipalities, there is a statistical difference (*p* < 0.0001), with a higher proportion of treatment in the reference in urban.

**Table 2:** Distribution of leprosy actions by comparison between the classifications of municipalities. Brazil. 2017–2018

Criteria	BRAZIL 37,342	RR^a^ 1,151	AR^b^ 11,190	RI^c^ 326	IA^d^ 3,752	Urban 20,923	*p*-value X^2^
	n (%)	n (%)	n (%)	n (%)	n (%)	n (%)	
When there is a person diagnosed with leprosy, the team:							
Conduct consultation in the unit itself	27,200 (72.8)	940 (81.7)	8,878 (79.3)	267 (81.9)	2,949 (78.6)	14,166 (67.7)	
Refers the person to the reference unit	10,142 (27.2)	211 (18.3)	2,312 (20.7)	59 (18.1)	803 (21.4)	6,757 (32.3)	
* p*-value		0.0907		0.3472	0.2181	< 0.0001	< 0.0001
Does the primary care team have a record of the number of people with leprosy?	27,957 (74.9)	849 (73.8)	8,175 (73.1)	267 (81.9)	2,824 (75.3)	15,842 (75.7)	
* p*-value		0.5992		0.0003	0.0173	< 0.0001	< 0.0001
Does the team request bacilloscopy for leprosy to be performed in the health services network?	35,717 (95.6)	1,079 (93.7)	10,710 (95.7)	313 (96)	3,608 (96.2)	20,007 (95.6)	
* p*-value		0.0019		0.6717	0.1007	0.7786	0.0132

Source: prepared by the authors. based on data from Program for the Improvement of Access and Quality of Primary Care (2019) and IBGE (2017)

^a^*RR* Rural Remote

^b^*AR* Adjacent Rural

^c^*RI* Remote Intermediary

^d^*IA* Intermediate Adjacent

There was no statistically significant difference in the action of having a record of the number of people with leprosy between remote rural and adjacent rural municipalities (*p* = 0.5992), but there was when comparing the other classifications, with a higher proportion in these. The action of requesting bacilloscopy for leprosy to be carried out in the network of health services in remote rural and adjacent rural municipalities showed a statistically significant difference (*p* = 0.0019), with a lower proportion in remote rural municipalities. When comparing the others, there was no statistical difference.

Table 3 shows the leprosy actions carried out by the primary health care teams in the unit itself. There was no statistically significant difference between remote rural and adjacent rural municipalities (*p* = 0.5992) in the diagnosis of new cases of leprosy, but there was when rural and urban municipalities were compared (*p* = 0.0004), with a higher proportion of diagnoses being made in rural. There was no statistically significant difference between the municipalities in the action of notifying people diagnosed with leprosy (*p* = 0.0951). As for the coordination of the person's care in the health network, for reference, it was evident that there is a statistically significant difference between rural municipalities, with a greater proportion in remote rural areas of teams that monitor the person with leprosy who is referred to the reference (*p*. = 0.0057).

**Table 3:** Distribution of leprosy actions by comparison between the classifications of municipalities, according to the teams that carry out consultations in primary health care. Brazil. 2017–2018

Teams that carry out consultations at the Unit	BRAZIL 27.200	RR^a^ 940	AR^b^ 8,878	RI^c^ 267	IA^d^ 2,949	Urban 14,166	*p*-value
	n (%)	n (%)	n (%)	n (%)	n (%)	n (%)	X^2^
Do you diagnose new cases of leprosy?	25,948 (69.5)	897 (95.4)	8,513 (95.9)	255 (95.5)	2,830 (96)	13,453 (95)	
*p*-value		0.5193		0.7944	0.7681	0.0004	0.0111
Do you notify the leprosy diagnoses made in the unit?	26,508 (71.0)	906 (96.4)	8,670 (97.7)	261 (97.8)	2,883 (97.8)	13,788 (97.3)	
*p*-value		0.0183		0.8236	0.5025	0.1749	0.0951
Do you monitor a person referred to reference health services?	26,624 (71.3)	906 (96.4)	8,678 (97.8)	263 (98.5)	2,896 (98.2)	13,881 (98)	
*p*-value		0.0057		0.3215	0.0619	0.2065	0.0083
The team performs an active search for the following cases:							
Symptomatic (skin lesions)	26,511 (71.0)	911 (96.9)	8,695 (97.95)	260 (97.4)	2,903 (98.4)	13,742 (97)	
* p*-value		0.0575		0.6352	0.0629	< 0.0001	< 0.0001
contact persons	26,631 (71.3)	918 (97.7)	8,693 (97.9)	259 (97)	2,900 (98.3)	13,861 (97.8)	
* p*-value		0.6011		0.3172	0.1165	0.4627	0.3698
Treatment abandonment	25,996 (69.6)	889 (94.6)	8,445 (95.1)	254 (95.1)	2,827 (95.9)	13,581 (95.9)	
* p*-value		0.437		0.962	0.0662	0.0131	0.0381

Source: prepared by the authors. based on data from Program for the Improvement of Access and Quality of Primary Care (2019) and IBGE (2017)

^a^*RR* Rural Remote

^b^*AR* Adjacent Rural

^c^*RI* Remote Intermediary

^d^*IA* Intermediate Adjacent

The active search for symptomatic people showed no statistical difference between rural municipalities (*p* = 0.0575). However, there was a difference when comparing this action carried out by the urban teams (*p* < 0.0001), with the rural presenting a higher proportion. There was no difference in the proportion performing the active search of leprosy contacts (*p* = 0.3698). The active search for treatment abandonment showed no statistical difference between rural municipalities, but when compared to urban ones, there was a statistically significant difference (*p* = 0.0131), with a greater proportion of action in urban.

Table 4 shows the actions of tuberculosis among the municipalities. No statistically significant difference was demonstrated in relation to the place of diagnosis of tuberculosis between rural municipalities, but when comparing the proportions of adjacent intermediaries (*p* = 0.0099) and urban (*p* < 0.0001) a significant difference was evidenced, with a higher proportion of teams that conduct the consultation at the unit itself in rural areas. Regarding the recording of the number of people with tuberculosis, there is a statistical difference between rural areas (*p* = 0.0005), with adjacent rural areas having a higher proportion of teams that register. As well as, a difference was evidenced when compared with the other classifications.

**Table 4:** Distribution of Tuberculosis actions by comparison between the classifications of municipalities. Brazil. 2017–2018

Criteria	Brazil 37,342	RR^a^ 1,151	AR^b^ 11,190	RI^c^ 326	IA^d^ 3,752	Urban 20,923	*p*-value X^2^
	n (%)	n (%)	n (%)	n (%)	n (%)	n (%)	
When there is a person diagnosed with Tuberculosis, the team:							
Conduct consultation in the unit itself	30,847 (82.6)	970 (84.3)	9,458 (84.5)	278 (85.3)	3,103 (82.7)	17,038 (81.4)	
Refers the person to the reference unit	6,495 (17.4)	181 (15.7)	1,732 (15.5)	48 (14.7)	649 (17.3)	3,885 (18.6)	
* p*-value		0.8333		0.7148	0.0099	< 0.0001	< 0.0001
Does the primary care team have a record of the number of people with tuberculosis?	31,239 (83.7)	859 (74.6)	8,794 (78.6)	269 (82.5)	3,006 (80.1)	18,311 (87.5)	
* p*-value		0.0005		0.0384	0.0093	< 0.0001	< 0.0001
Which of these tests does the team request to be performed in the health services network?							
Sputum smear microscopy	36,674 (98.2)	1,112 (96.6)	10,981 (98.1)	316 (96.9)	3,704 (98.7)	20,561 (98.3)	
* p*-value		0.0002		0.1549	0.0021	0.3339	< 0.0001
Chest X-ray	36,718 (98.3)	1,107 (96.2)	10,953 (97.9)	315 (96.6)	3,714 (99)	20,629 (98.6)	
* p*-value		< 0.0001		0.1271	< 0.0001	< 0.0001	< 0.0001

Source: prepared by the authors. based on data from Program for the Improvement of Access and Quality of Primary Care (2019) and IBGE (2017)

^a^*RR* Rural Remote

^b^*AR* Adjacent Rural

^c^*RI* Remote Intermediary

^d^*IA* Intermediate Adjacent

Regarding the request for tests to be carried out in the health care network. The request for Sputum smear microscopy for tuberculosis by the teams showed a statistical difference between rural people (*p* = 0.0002), with a lower proportion of requests in remote rural areas. When compared to adjacent intermediate municipalities, a statistical difference was demonstrated (*p* = 0.0021), with a higher proportion of requests in adjacent intermediaries. The results demonstrate a statistically significant difference in the request for chest X-rays among rural people (*p* < 0.0001), with a smaller proportion in remote rural areas. When compared to adjacent intermediaries (*p* < 0.0001) and urban (*p* < 0.0001), a statistically significant difference is evident, with these municipalities presenting a higher proportion in relation to rural.

Table 5 shows the prevention, diagnosis and treatment of tuberculosis carried out by the teams at the health unit itself. As for the 1st Sputum smear microscopy for the diagnosis of tuberculosis being collected in the first approach/appointment, there was a statistical difference between the rural classification (*p* < 0.0001), with the adjacent rural areas presenting a higher proportion of collection in the first appointment. There was a statistical difference in this action carried out in rural areas when compared to remote intermediaries (*p* < 0.0001) and urban (*p* < 0.0001), with a higher proportion in rural areas in relation to remote intermediaries and a smaller proportion in relation to urban.

**Table 5:** Distribution of tuberculosis actions by comparison between the classification of municipalities, according to the teams that carry out consultations in primary health care. Brazil. 2017–2018

Teams that carry out consultations at the Unit	Brazil 30,847	RR^a^ 970	AR^b^ 9,458	RI^c^ 278	IA^d^ 3,103	Urban 17,038	*p*-value X^2^
	n (%)	n (%)	n (%)	n (%)	n (%)	n (%)	
Is the 1st Sputum smear microscopy for the diagnosis of tuberculosis collected at the first approach/consultation?	21,830 (70.8)	567 (58.5)	6,363 (67.3)	153 (55.0)	2.041 (65.8)	12,706 (74.6)	
*p*-value		< 0.0001		< 0.0001	0.6787	< 0.0001	< 0.0001
Does the team notify people with tuberculosis diagnosed in the unit?	30,055 (97.4)	925 (95.4)	9,151 (96.8)	272 (97.8)	3,017 (97.2)	16,690 (98.0)	
*p*-value		0.009		0.2054	0.0759	< 0.0001	< 0.0001
Does the team monitor the treatment directly observed by the person?	29,885 (96.9)	910 (93.8)	9,212 (97.4)	262 (94.2)	3,019 (97.3)	16,482 (96.7)	
*p*-value		< 0.0001		0.0076	0.3963	0.1044	< 0.0001
The team performs an active search for the following cases:							
respiratory symptoms	29,827 (96.7)	915 (94.3)	9,195 (97.2)	261 (93.9)	3,017 (97.2)	16,439 (96.5)	
* p*-value		< 0.0001		0.0048	0.3267	0.0226	< 0.0001
contact person	30,277 (98.2)	932 (96.1)	9,271 (98)	265 (95.3)	3,049 (98.3)	16,760 (98.4)	
* p*-value		< 0.0001		0.0021	0.0787	0.0017	< 0.0001
person lost to follow-up (after the 30-day period)	29,574 (95.9)	900 (92.8)	8,993 (95.1)	264 (95)	2,963 (95.5)	16,454 (96.6)	
* p*-value		0.0006		0.9377	0.1286	< 0.0001	< 0.0001

Source: prepared by the authors. based on data from Program for the Improvement of Access and Quality of Primary Care (2019) and IBGE (2017)

^a^*RR* Rural Remote

^b^*AR* Adjacent Rural

^c^*RI* Remote Intermediary

^d^*IA* Intermediate Adjacent

Regarding the notification of people with tuberculosis diagnosed in the unit, there is a significant difference between rural areas (*p* = 0.009), with a higher proportion of teams reporting in adjacent rural areas. When compared to urban areas, there is a statistical difference (*p* < 0.0001) with a higher proportion of notifications in urban areas compared to rural areas.

As for directly observed treatment monitoring, there is a significant difference between rural areas (*p* < 0.0001), with a higher proportion of teams carrying out monitoring in adjacent rural areas. When compared to remote intermediaries, there was a statistically significant difference (*p* = 0.0076), with a higher proportion being observed in rural areas.

In the active search actions of people with tuberculosis, there is a statistical difference between rural people in the active search for respiratory symptoms (*p* < 0.0001), contact people (*p* < 0.0001), people lost to follow-up (*p* = 0.0006), with rural people adjacent areas with a higher proportion of teams carrying out these actions. Comparing the active search for respiratory symptoms carried out in rural and intermediate remote (*p* = 0.0048) and urban (*p* = 0.0226) municipalities, significant statistics were found, with both classifications having a lower proportion of active search in relation to rural areas. There was a statistical difference in the active search for contact persons carried out by the teams, when comparing the rural to the remote intermediary (*p* = 0.0021) and urban (*p* = 0.0017), with a lower proportion in the intermediate and higher in the urban. Regarding the active search for treatment abandonment, there is a statistical difference between the action taken in rural areas when compared to urban areas (*p* < 0.0001), with a higher proportion of teams carrying out this active search in urban municipalities.

## Discussion

The study analyzed data from tuberculosis and leprosy actions of diagnosis and treatment carried out by Primary Health Care teams in rural and urban areas in Brazil. As well as analyzing the data of the tests that are requested by the teams to be carried out in the health services network, from the Program for the Improvement of Access and Quality of Primary Care. In the country, it is observed that even after years of the implementation of the Leprosy Control Program and the implementation of the health care network that led to changes in the organization of health services, obstacles still persist that hinder access and weaken the program [6–9, 11] and, consequently, may have an impact on disease indicators. Urban municipalities have a higher proportion of teams that do not carry out leprosy actions of diagnosis and treatment in the unit itself (a higher proportion of treatment in the reference and low proportion of teams that diagnose new cases of leprosy) in urban. Demonstrating problems in access to Primary Health Care, comprehensiveness and coordination of care. This situation and other factors may contribute to the high incidence that occurs in urban municipalities [15]. This higher number of teams that refer the person to the reference can mean a high number of people with a complex diagnosis or lack of training of the teams to carry out the diagnosis in the unit itself.

In leprosy actions of diagnosis and treatment, less than 90% of the teams in all types of municipalities carry out consultations with the user diagnosed at the Basic Health Unit itself, with this situation being more critical in municipalities classified as urban. Thus, there are implications for reducing the population's access to early diagnosis, with greater use of specialized services [8, 9]. The results of the present study also show that there is a deficiency in the coordination of care in municipalities classified as remote rural areas, since it is the typology with the highest proportion of teams that do not request bacilloscopy to be performed in the network of services and that do not monitor person referred to reference health services. In a municipality classified as intermediate adjacent in the North region, materials for the clinical diagnosis of new cases of leprosy are available in 100% of the Basic Health Units [7]. In surveillance actions, failures in active search actions were verified, which were also evidenced in municipalities in the North and Northeast regions, in which active search, when performed improperly, increases the chances of transmission and illness in the family network and guarantees the maintenance of the disease between generations [9].

The fragmentation of care in leprosy actions of diagnosis and treatment, by Primary Health Care teams was also observed in all types of municipalities. The results of this study corroborate the literature regarding the difficulties in diagnosis, timely treatment and follow-up of people affected by leprosy [9, 10]. In general, the results showed inequalities in carrying out actions, according to the characteristics of the territories. As demonstrated in the study, these problems also occur in urban municipalities that have an incipient degree of implementation of the program, with a low proportion of surveillance actions of examined contacts and treatment abandonment, limited standardization of the service flow to person and poor resolution of obstacles by the management [23]. It appears that the Primary Health Care teams still exercise their assistance in a reactive way, when sought by person with assistance limited to administering the supervised dose without an expanded evaluation of the user [9].

All care actions for people with Tuberculosis were significantly associated with some type of municipality and the greatest inequalities were observed, mainly in rural areas and remote intermediaries. A study carried out with data from the first cycle of the Program for the Improvement of Access and Quality of Primary Care also found an association with the population size of the municipalities in the actions studied. The highest percentages of teams carrying out active search actions, requesting bacilloscopy, having a notification form and administration of the daily dose of medication by a health professional to the user (the directly observed treatment) were in the metropolises. While small municipalities had the lowest percentages of active search [18]. However, a study carried out in Rio Grande do Sul in a remote rural municipality found better results related to continuity of care and case detection [24].

As with leprosy actions of diagnosis and treatment, in urban municipalities there is less access to consultations in the unit itself. This result corroborates what was found in urban municipalities in the South and North regions from Brazil [23, 25]. this context, Primary Health Care assumes the role of regulating the flow, with the main actions attributed to Primary Health Care services being delegated to specialized care [23, 25]. Tuberculosis actions of diagnosis and treatment when carried out in Primary Health Care units show better results, as the treatment is carried out in the Basic Health Unit closest to home, there is less loss of the day work or appointments for the Tuberculosis consultation and lower expenses with transportation. Directly observed treatment is a strategy that makes it possible to build a professional-user bond [26], especially when performed in the person's home territory [25].

In Brazil, the North and Northeast regions have the highest percentages of new Tuberculosis cases notified and followed up in the Primary Health Care. In Pará, more than 80% were monitored in the Primary Health Care. Unlike Santa Catarina, Rio Grande do Sul and the Federal District, where less than 40% of cases were notified and followed up in the Primary Health Care, with new Tuberculosis cases being concentrated in other levels of care [4], and thus may have an impact on directly observed treatment. The inequalities of access to health in rural areas in relation to urban areas have been demonstrated in previous studies [27, 28]. The work process of the Family Health Strategy teams is more complex due to organizational issues such as lack of transportation, which is important due to the great distances and dispersion of populations in these municipalities [27]. The basic service package of the current National Primary Care Policy and the current financing model may further increase the inequalities of municipalities in relation to Tuberculosis and leprosy actions of diagnosis and treatment, since it implies the financing and proposes different modalities of Primary Health Care teams, whose work process does not contemplate the characteristics of the territory and collective health [10, 11, 17].

The study has limitations regarding the intentionality in the selection of teams that rehired, but the high number of teams reduces the selection bias. While in the database there was no classification of four municipalities, but it was minimized by the small number. The results suggest a reflection on the existing disparities between the regions of Brazil and the municipalities according to the current classification. It is important to reflect on the infrastructure and work process of the teams.

## Conclusions

The Primary Health Care plays a strategic role in the actions of diagnosis and treatment of leprosy and Tuberculosis. The results showed a greater number of teams that carry out consultations for Tuberculosis in the Basic Health Unit in relation to consultations for leprosy. There is deficiency in the diagnosis, treatment and surveillance actions related to the control actions of these diseases with marked inequalities between the typologies of the municipalities.

The study presents as contributions to the area data for an analysis of leprosy and tuberculosis actions of diagnosis and treatment, evidencing the indispensability of funding equity and differentiated management approaches according to municipal differences.

## Data Availability

Data is available online in the Secretaria de Atenção Primária à Saúde, platform with access provided by Ministry of Health https://aps.saude.gov.br/ape/pmaq/ciclo3/. The datasets used during this current study are also available from the corresponding author on reasonable request.
